# Impact of adjuvant radiotherapy on the survival of women with optimally resected stage III endometrial cancer in the era of modern radiotherapy: a retrospective study

**DOI:** 10.1186/s13014-020-01523-5

**Published:** 2020-04-06

**Authors:** Jenny Ling-Yu Chen, Yu-Sen Huang, Chao-Yuan Huang, Che-Yu Hsu, Keng-Hsueh Lan, Wen-Fang Cheng, Sung-Hsin Kuo

**Affiliations:** 1 Department of Radiology, National Taiwan University College of Medicine, No. 7, Chung-Shan S. Rd, Taipei, Taiwan; 2 Division of Radiation Oncology, Department of Oncology, National Taiwan University Hospital, Taipei, Taiwan; 3 Department of Medical Imaging, National Taiwan University Hospital Hsin-Chu Branch, Hsinchu, Taiwan; 4 National Taiwan University Cancer Center, National Taiwan University College of Medicine, Taipei, Taiwan; 5 Department of Medical Imaging, National Taiwan University Hospital, Taipei, Taiwan; 6 Department of Medical Imaging, National Taiwan University Hospital Yun-Lin Branch, Yunlin, Taiwan; 7 Department of Obstetrics and Gynecology, National Taiwan University Hospital, Taipei, Taiwan

**Keywords:** Endometrial cancer, Stage III, Adjuvant therapy, Intensity-modulated radiotherapy, Volumetric modulated arc radiotherapy

## Abstract

**Background:**

The optimal adjuvant treatment for stage III endometrial cancer in the era of modern radiotherapy remains undefined. We investigated the benefit of adjuvant radiotherapy for women who underwent optimal resection for stage III endometrial cancer in the era of modern radiotherapy.

**Methods:**

We retrospectively reviewed patients with endometrial cancer who were treated between 2010 and 2018. Adjuvant treatment included radiotherapy by modern radiotherapy techniques (intensity-modulated or volumetric modulated arc radiotherapy), chemotherapy, or both. Recurrence-free survival (RFS) and overall survival (OS) were calculated using the Kaplan-Meier method and analyzed via multivariate Cox proportional hazards models.

**Results:**

One hundred sixty-one patients were initially included (52, 9, and 100 with stages IIIA, IIIB, and IIIC cancer, respectively); 154 patients (96%) received adjuvant therapy. Such adjuvant treatment was associated with improved RFS (*p* = 0.014) and OS (*p* = 0.044) over surgery alone. Adjuvant radiotherapy by modern radiotherapy techniques led to low incidence of acute (25%) and chronic (7%) grade ≥ 2 gastrointestinal toxicity. On univariate analysis, non-endometrioid histology and grade 3 status were associated with higher risks of tumor recurrence and death, whereas adjuvant radiotherapy alone or in combination chemotherapy reduced their risks. On multivariate analysis, non-endometrioid histology was associated with increased recurrence (hazard ratio [HR], 2.95; *p* = 0.009), whereas adjuvant radiotherapy alone or with chemotherapy was associated with lower recurrence (HR, 0.62; *p* = 0.042). Patients > 60 years of age (*p* = 0.038) as well as those with endometrioid histology (*p* = 0.045), lymphovascular space invasion (*p* = 0.031), and ≥ 2 positive lymph nodes (*p* = 0.044) benefited most from adjuvant radiotherapy.

**Conclusions:**

Modern adjuvant radiotherapy (intensity-modulated or volumetric modulated arc radiotherapy) alone or with chemotherapy should be considered for women with optimally resected stage III endometrial cancer.

**Trial registration:**

ClinicalTrials.gov, NCT04251676. Registered 24 January 2020. Retrospectively registered.

## Background

Endometrial cancer is the most common malignancy of the female genital tract; moreover, its incidence rate continues to increase [1]. Approximately one-fifth of the patients are diagnosed at an already advanced stage; the 5-year overall survival (OS) rates of those with stage III disease who are able to undergo optimal resection are 70–80% when adjuvant therapy is administered and 30–40% when it is not [2, 3]. The most suitable adjuvant treatment for stage III endometrial cancer remains undefined, including the appropriate adjuvant chemotherapy, radiotherapy, or combined chemoradiotherapy options [4–7]; notably, only a few prospective studies on this topic have been performed [8, 9].

Adjuvant external beam radiotherapy (EBRT) is conventionally delivered using multiple conformal fields via three-dimensional conformal radiation therapy; modern radiotherapy techniques such as intensity-modulated radiation therapy (IMRT) and volumetric modulated arc therapy (VMAT) have reduced treatment-related toxicity and are becoming more widely available [10, 11]. IMRT, which has the advantage of integrating a multileaf collimator, rotational fan, cone beam delivery system, and robotic arm linear accelerator, delivers a high radiation dose to the target while minimizing the exposure of the organs at risk. Moreover, the arc-based approach for VMAT delivery is designed to further improve dose distribution through dynamic modulation of the gantry rotation speed, dose rate, and multileaf collimator shaping, thereby generating patterns of intensity modulation that deliver optimal treatment doses to the patient [12, 13]. Modern radiotherapy techniques are associated with better survival rates than conventional radiation delivery for numerous cancer treatments [14, 15]. This is mostly due to their qualities of risk-adaptive dose prescription, increased locoregional tumor control, and decreased radiation-related side effects [16, 17]. Yet, the merits of modern radiotherapy techniques for adjuvant endometrial cancer are still being investigated.

## Materials and methods

The aim of this study was to investigate the benefit of adjuvant radiotherapy for women who underwent optimal resection for stage III endometrial cancer in the era of modern radiotherapy.

### Study design and patient selection

Patients with surgically staged endometrial cancer treated between 2010 and 2018 within the multi-institution National Taiwan University Hospital Healthcare System (including the National Taiwan University Hospital, the affiliated Yun-Lin branch, and Hsin-Chu branch) were retrospectively investigated. Staging was performed according to the 2009 International Federation of Gynecology and Obstetrics (FIGO) staging system for endometrial cancer [18]. A total of 1273 patients with surgically staged endometrial cancer were identified, among whom 204 had FIGO stage III. Women who were administered chemotherapy or radiotherapy before surgery, received surgery with a palliative intent, or had sarcoma or carcinosarcoma were excluded; hence, 161 patients with stage III endometrial cancer who underwent optimal resection were ultimately included.

### Surgery

Staging surgery included total abdominal hysterectomy, bilateral salpingo-oophorectomy, peritoneal washings, and either selective or systematic pelvic lymphadenectomy. The para-aortic lymph nodes were sampled or dissected from the area between the aortic bifurcation and inferior mesenteric artery in patients with elevated serum cancer antigen 125, myometrial invasion > 50%, extrauterine spread, or para-aortic lymph nodes > 1 cm in diameter as identified on preoperative magnetic resonance imaging (per the Korean Gynecologic Oncology Group 2014 criteria). Bulky nodes were removed by dissection whenever possible. An omental biopsy or omentectomy was performed at the discretion of the individual surgeon based on the extent of disease. The patients had no residual disease by the end of primary surgery; the median number of dissected lymph nodes was 12 (range: 0–40).

### Chemotherapy

Chemotherapy regimens were administered according to the physicians’ preferences; the most common treatments were platinum-based regimens with the majority (81%) receiving paclitaxel and platinum (carboplatin or cisplatin). Other regimens included cisplatin plus doxorubicin (10%), and cisplatin plus epirubicin (9%). The majority of patients received 6 cycles of chemotherapy. Women who received both adjuvant chemotherapy and radiotherapy completed all chemotherapy courses before radiotherapy (34%) or in conjunction with radiotherapy in a sandwich pattern (66%).

### Radiotherapy

**Fig. 1:**
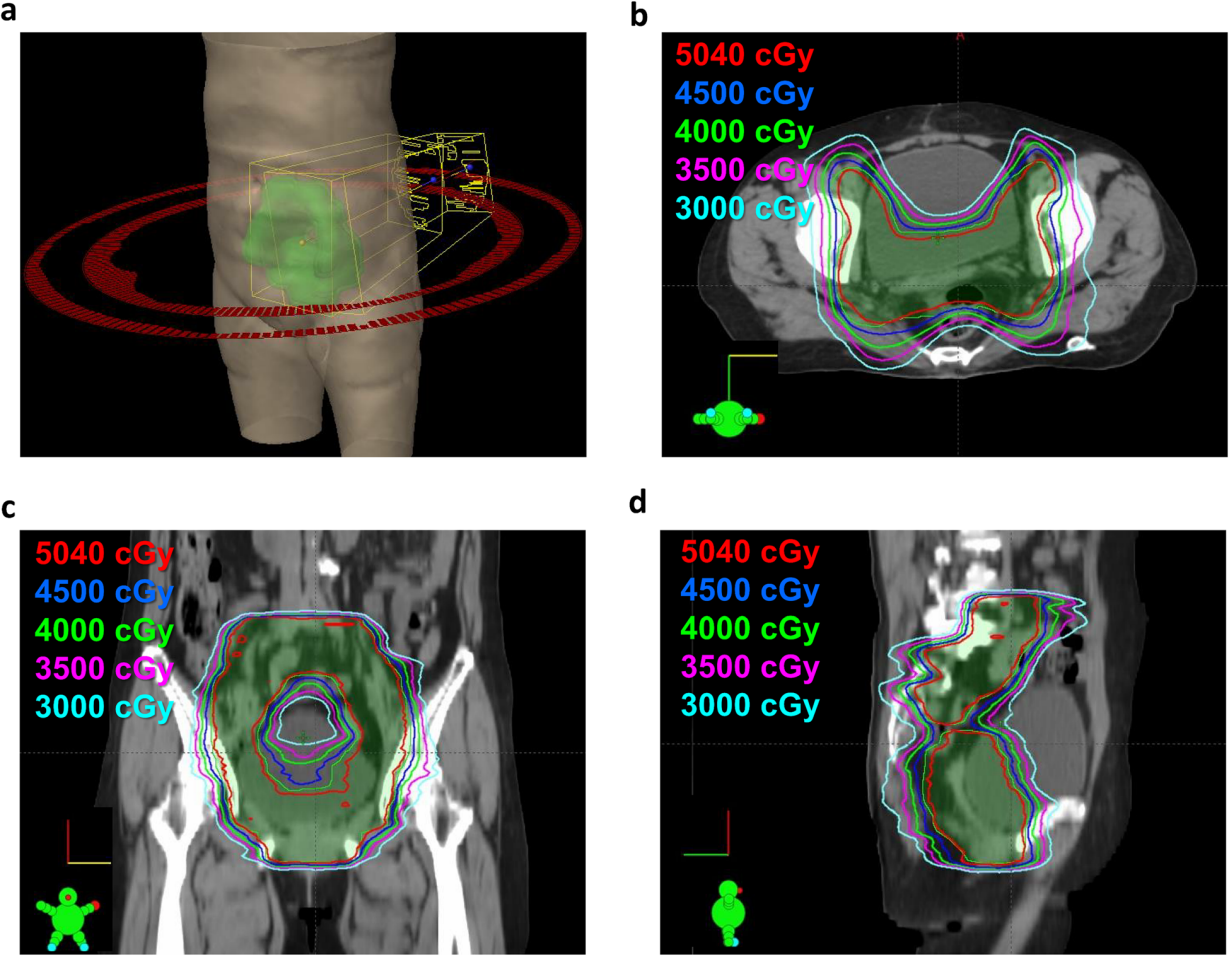
Modern radiotherapy technique and dose distributions. This figure shows the isodose distributions in a patient with optimally resected stage III endometrial cancer who underwent adjuvant radiotherapy via volumetric modulated arc therapy (VMAT). A 50.4-Gy dose (28 fractions) was prescribed to the target volumes. **a** Beam arrangement according to the VMAT plan. Dose distributions in the axial (**b**), coronal (**c**), and sagittal (**d**) views. The green color-washed areas indicate the target volume (i.e., vagina and nodal lymphatics in the pelvis). The red, blue, green, pink, and indigo lines represent isodose curves of 50.4, 45, 40, 35, and 30 Gy, respectively.

For optimally resected stage III endometrial cancer, the adjuvant radiotherapy was directed at sites of known or suspected tumor involvement, and included EBRT and/or brachytherapy [19]. All women who underwent adjuvant radiotherapy were treated with EBRT, and vaginal brachytherapy was also used for most (84%) women. Our health-care system adopted modern radiotherapy including IMRT since 2010 and VMAT since 2015, which helped minimize the dose to the normal organs on the basis of adjuvant radiotherapy [20, 21]. The dose of EBRT was 5040 cGy over 6 weeks, 5 days per week, with daily fractions of 1.8 Gy. Pelvic radiotherapy targeted the common, external, and internal iliac lymph node regions, upper 3 cm of the vagina, and the paravaginal soft tissue lateral to the vagina in accordance with the updated delineation consensus for gynecologic malignancy [11]. The presacral lymph nodes were irradiated in patients with cervical involvement. In patients with multiple positive pelvic nodes or documented para-aortic lymph node disease, extended-field radiotherapy that encompassed the pelvic volume and also targeted the entire common iliac chain and para-aortic lymph node region was considered. A boost dose of 5–10 Gy was also considered for documented extranodal extension or enlarged unresected lymphadenopathy. An example of a modern radiotherapy technique with its associated isodose curves is shown in Fig. 1. EBRT was performed using 10 MV radiation beams from the Elekta Synergy accelerator (Elekta, Stockholm, Sweden) or the Varian TrueBeam™ Radiotherapy System (Varian, Palo Alto, CA, USA) in multiple coplanar ports. The treatment position was verified weekly using cone-beam CT X-ray volume imaging [14]. Following pelvic radiation, high dose-rate brachytherapy via a vaginal cylinder was used to boost the upper two-thirds of the vagina. Brachytherapy doses of 6 Gy per fraction for 2 to 3 fractions prescribed to the vaginal mucosa were used, and they were delivered using the “Nucletron HDR” Ir-192 remote afterloading technique [22, 23].

### Statistical analysis

Statistical analyses were performed using the Statistical Package for Social Sciences for Windows, version 22.0 (IBM, Armonk, NY, USA). Differences in the distributions of variables among the treatment groups were analyzed using the chi-squared test. Acute and late toxicities were rated according to the Common Terminology Criteria for Adverse Events version 4.0. Survival data were confirmed with the Cancer Registry Medical Information Management Office in the health-care system. All patients were followed for at least 3 months for the first 2 years and every 4–6 months thereafter until recurrence or death [2, 23]. Locoregional recurrence was defined as failure in the pelvic regions or evidence of para-aortic lymphadenopathy below the T12–L1 interspace. Distant metastasis was defined as disease relapse outside the locoregional area as detected pathologically, cytologically, or radiologically. All events were calculated from the date of treatment completion. Analysis was conducted using the follow-up data accumulated as of September 30, 2019. Actuarial estimates of recurrence-free survival (RFS) and OS were calculated using the Kaplan-Meier method and compared using the log-rank test. All prognostic variables found to be significant on univariate analysis were subjected to multivariate analysis using the Cox proportional hazards regression model. A *p*-value ≤0.05 was considered statistically significant.

## Results

### Patient characteristics

A total 161 optimally resected patients were investigated (52 with stage IIIA, 9 with stage IIIB, and 100 with stage IIIC disease); none had residual disease upon completing primary surgery. The patient and tumor characteristics are shown in Table 1. The median age at diagnosis was 57 years, and the predominant histologic subtype was endometrioid (85%). Lymphovascular space invasion was present in nearly three-fourths (74%) of the tumors, and nearly one-third (32%) had FIGO grade 3 histology. Positive peritoneal washing cytology was observed in nearly one-fourth (24%) of the patients.

**Table 1:** Patients’ demographics and tumor characteristics (*n* = 161)

Age (years) [range]	57 [33–89]
FIGO stage	
IIIA	52 (32)
IIIB	9 (6)
IIIC	100 (62)
Histology	
Endometrioid	137 (85)
Papillary serous	16 (10)
Clear cell	5 (3)
Mucinous	2 (1)
Neuroendocrine	1 (1)
Tumor grade	
1	52 (32)
2	57 (36)
3	52 (32)
Lymphovascular space invasion	
Nil	42 (26)
Present	119 (74)
Peritoneal cytology	
Negative	122 (76)
Positive	39 (24)
Pelvic LN metastasis	
Nil	66 (41)
Present	95 (59)
Median number of positive LN [range]	2 [1–11]
Para-aortic LN metastasis	
Nil	135 (84)
Present	26 (16)
Median number of positive LN [range]	2 [1–5]
Total LN metastasis	
Nil	61 (38)
Present	100 (62)
Median number of positive LN [range]	2 [1–12]

Values are presented as median [range] or n (%)

*FIGO* The International Federation of Gynecology and Obstetrics, *LN* lymph node

### Adjuvant treatments and outcomes

One hundred fifty-four patients (96%) received an adjuvant treatment; 67 (42%) received chemotherapy alone, 29 (18%) received adjuvant radiotherapy alone, and 58 (36%) underwent chemoradiotherapy. In the 87 patients who received adjuvant radiotherapy with or without chemotherapy, IMRT was used in 45 (52%) patients and VMAT was used in 42 (48%) patients. Of the remaining 7 patients, 4 decided against adjuvant therapy and 3 had comorbidities that precluded such treatments. The relationships between the patients’ characteristics and type of postoperative adjuvant treatment received are described in Table 2. There was no significant difference in the type of administered treatment among patients grouped according to FIGO stage, tumor histology, tumor grade, lymphovascular space invasion, positive peritoneal cytology, or number of positive lymph nodes. However, elderly patients were less frequently treated with combined chemoradiotherapy than were their younger counterparts (*p* = 0.046).

**Table 2:** Relationship between treatment type and patient characteristics (*n* = 161)

	Chemotherapy (*n* = 67)	Radiotherapy (*n* = 29)	Combined chemoradiotherapy (*n* = 58)	No treatment (*n* = 7)	*p*-value^a^
Age					*0.046*
≤ 60 years	42 (41%)	14 (14%)	43 (42%)	4 (3%)	
> 60 years	25 (43%)	15 (26%)	15 (26%)	3 (5%)	
FIGO stage					*0.389*
IIIA or IIIB	30 (49%)	8 (13%)	20 (33%)	3 (5%)	
IIIC	37 (37%)	21 (21%)	38 (38%)	4 (4%)	
Histology					*0.059*
Endometrioid	56 (41%)	28 (20%)	49 (36%)	4 (3%)	
Non-endometrioid	11 (46%)	1 (4%)	9 (38%)	3 (12%)	
Tumor grade					*0.185*
1–2	46 (42%)	24 (22%)	35 (32%)	4 (4%)	
3	21 (40%)	5 (10%)	23 (44%)	3 (6%)	
Lymphovascular space invasion					*0.225*
Nil	22 (52%)	4 (10%)	15 (36%)	1 (2%)	
Present	45 (38%)	25 (21%)	43 (36%)	6 (5%)	
Peritoneal cytology					*0.111*
Negative	45 (367%)	23 (19%)	47 (39%)	7 (6%)	
Positive	22 (576%)	6 (15%)	11 (28%)	0 (0%)	
≥2 positive LN					*0.669*
Nil	38 (44%)	14 (16%)	30 (34%)	5 (6%)	
Present	29 (39%)	15 (20%)	28 (38%)	2 (3%)	

*FIGO* The International Federation of Gynecology and Obstetrics, *LN* lymph node

^a^Significance was determined using the chi-squared test

**Fig. 2:**
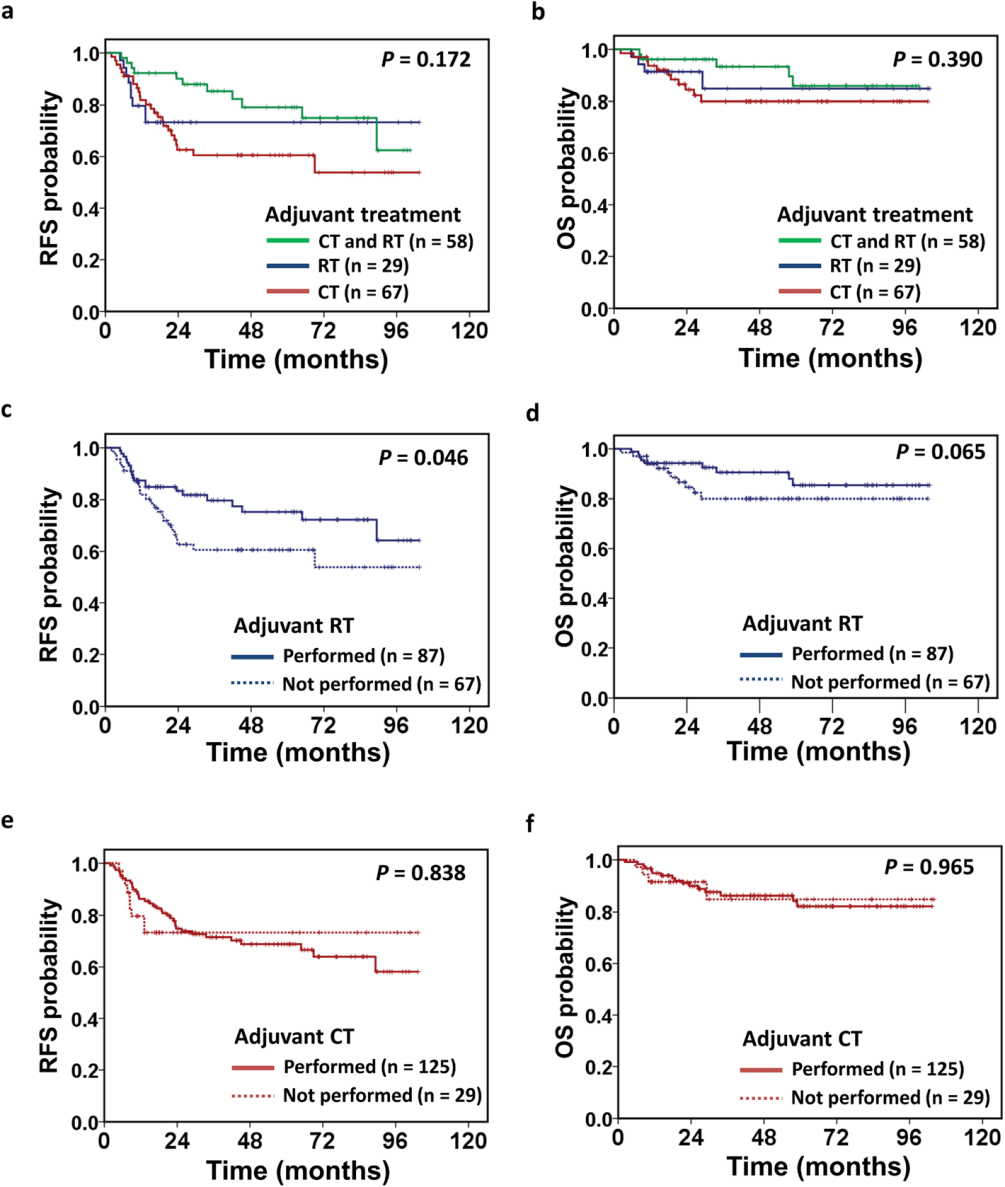
Survival in patients with optimally resected stage III endometrial cancer by type of adjuvant treatment (*n* = 154). Recurrence-free survival (RFS) (**a**) and overall survival (OS) (**b**) of patients based on the type of adjuvant treatment: adjuvant chemotherapy (CT) alone, adjuvant radiotherapy (RT) alone, or combined chemoradiotherapy. RFS (**c**) and OS (**d**) of patients based on whether or not they received radiotherapy alone or combined with chemotherapy. RFS (**e**) and OS (**f**) of patients based on whether or not they received chemotherapy alone or combined with radiotherapy. *p*-values were determined using Kaplan-Meier log-rank tests.

Regarding adjuvant radiotherapy-related gastrointestinal side effects, 23% of patients had acute grade 2 diarrhea, and 2% had acute grade 3 diarrhea; 5% had grade 2 late toxicity with moderate diarrhea requiring medications, and 2% developed grade 3 radiation proctitis that were successfully treated by colonoscopy argon plasma coagulation. None had acute or late grade 4+ gastrointestinal symptoms. After a median follow-up of 44 months (range: 3–103 months), 140 patients (87%) were alive, whereas 21 patients (13%) had died; the majority of deaths (95%) were attributed to cancer progression. Fifty-one patients had tumor recurrence, which was locoregional in 26 (vaginal stump, pelvic, or para-aortic recurrence) and distant in 41 (lung, liver, bone, brain, distant lymphadenopathy, or peritoneal carcinomatosis); moreover, 16 patients had both locoregional recurrence and distant metastasis. Administration of any type of adjuvant treatment was associated with a longer 5-year RFS (67% vs. 0%, *p* = 0.014, Supplementary Figure 1a) and OS (84% vs. 66%, *p* = 0.044, Supplementary Figure 1b). The type of adjuvant treatment did not influence the risks of tumor recurrence in patients with optimally resected stage III disease; the 5-year RFS was 61% in those who underwent adjuvant chemotherapy alone, 73% in those who received adjuvant radiotherapy alone, and 79% in those who received combined chemoradiotherapy (*p* = 0.172, Fig. 2a). Likewise, the 5-year OS was 80% for patients treated with adjuvant chemotherapy alone, 85% for those treated with adjuvant radiotherapy alone, and 86% for those who received combined chemoradiotherapy (*p* = 0.390, Fig. 2b).

On the univariate analysis, tumor stage (IIIA or IIIB vs. IIIC), presence of lymphovascular space invasion, positive peritoneal washing cytology, or ≥ 2 positive lymph nodes were not significantly associated with an increased risk of tumor recurrence or death, whereas older age was associated with an increased risk of tumor recurrence (hazard ratio [HR] 2.14, *p* = 0.008) but not of death (Table 3). Non-endometrioid histology and grade 3 tumor status were associated with increased risks of tumor recurrence (HR: non-endometrioid histology: 5.42; grade 3 tumor status: 1.74, both *p* < 0.001) and death (HR: non-endometrioid histology: 4.34, *p* = 0.001; grade 3 tumor status: 1.49, *p* = 0.043).

**Table 3:** Univariate analysis of potential prognostic factors (*n* = 154)

	5-year RFS	HR (95% CI)	*p*-value^a^	5-year OS	HR (95% CI)	*p*-value^a^
Age			*0.008*			*0.459*
≤ 60 years	76	–		86	–	
> 60 years	52	2.14 (1.20–3.79)		81	1.41 (0.57–3.51)	
FIGO stage			*0.451*			*0.615*
IIIA or IIIB	69	–		85	–	
IIIC	66	0.89 (0.65–1.21)		84	0.88 (0.55–1.43)	
Histology			*< 0.001*			*0.001*
Endometrioid	75	–		87	–	
Non-endometrioid	15	5.42 (2.88–10.18)		62	4.34 (1.63–11.60)	
Tumor grade			*< 0.001*			*0.043*
1–2	78	–		88	–	
3	44	1.74 (1.30–2.32)		76	1.49 (1.05–2.35)	
Lymphovascular space invasion			*0.950*			*0.232*
Nil	69	–		87	–	
Present	64	1.01 (0.71–1.37)		76	1.32 (0.83–2.11)	
Peritoneal cytology			*0.174*			*0.316*
Negative	70	–		86	–	
Positive	58	1.54 (0.82–2.89)		75	1.64 (0.62–4.33)	
≥2 positive LN			*0.545*			*0.316*
Nil	69	–		87	–	
Present	66	1.19 (0.67–2.12)		82	1.59 (0.64–3.95)	
Adjuvant radiotherapy, alone or combined with chemotherapy			*0.046*			*0.065*
Yes	75	0.62 (0.35–0.99)		85	0.53 (0.21–1.23)	
No	61	–		80	–	
Adjuvant chemotherapy, alone or combined with radiotherapy			*0.838*			*0.965*
Yes	69	1.08 (0.52–2.25)		82	1.03 (0.34–3.07)	
No	73	–		85	–	
Combined chemoradiotherapy			*0.084*			*0.522*
Yes	81	0.48 (0.20–1.13)		87	0.67 (0.19–2.31)	
No	64	–		84	–	

*FIGO* The International Federation of Gynecology and Obstetrics, *LN* lymph node, *RFS* recurrence-free survival, *OS* overall survival, *HR* hazard ratio, *CI* confidence interval

^a^Significance tested using Kaplan–Meier life table analysis and the log-rank test

Moreover, the type of adjuvant treatment was associated with clinical outcomes. Patients who received adjuvant radiotherapy alone or combined with chemotherapy experienced a longer 5-year RFS than those who did not receive radiotherapy (75% vs. 61%, *p* = 0.046, Fig. 2c) and also showed a trend toward a longer 5-year OS rate (85% vs. 80%, *p* = 0.065, Fig. 2d). Meanwhile, patients who received adjuvant chemotherapy alone or combined with radiotherapy had similar survival periods as those who did not receive chemotherapy (5-year RFS: 69% vs. 73%, *p* = 0.838, Fig. 2e; 5-year OS: 82% vs. 85%, *p* = 0.965, Fig. 2f).

On the multivariate analysis, non-endometrioid histology increased the risk of tumor recurrence (HR 2.95, 95% confidence interval [CI] 1.32–6.61, *p* = 0.009), whereas adjuvant radiotherapy alone or combined with chemotherapy decreased the risk of tumor recurrence (HR 0.62, 95% CI 0.31–0.98, *p* = 0.042) (Table 4).

**Table 4:** Multivariate analysis of potential prognostic factors (*n* = 154)

	Recurrence-free survival		Overall survival	
	HR (95% CI)	*p*-value^a^	HR (95% CI)	*p*-value^a^
Age > 60 years	1.44 (0.75–2.75)	*0.277*	0.93 (0.34–2.56)	*0.884*
Non-endometrioid histology	2.95 (1.32–6.61)	*0.009*	3.27 (0.94–11.34)	*0.062*
Tumor grade 3	1.90 (0.97–3.75)	*0.062*	1.38 (0.48–3.98)	*0.555*
Adjuvant radiotherapy, alone or combined with chemotherapy	0.62 (0.31–0.98)	*0.042*	0.53 (0.20–1.37)	*0.188*

*HR* hazard ratio, *CI* confidence interval

^a^Significance was tested using multivariate analysis based on the Cox proportional hazards regression model

### Effectiveness of adjuvant radiotherapy

**Fig. 3:**
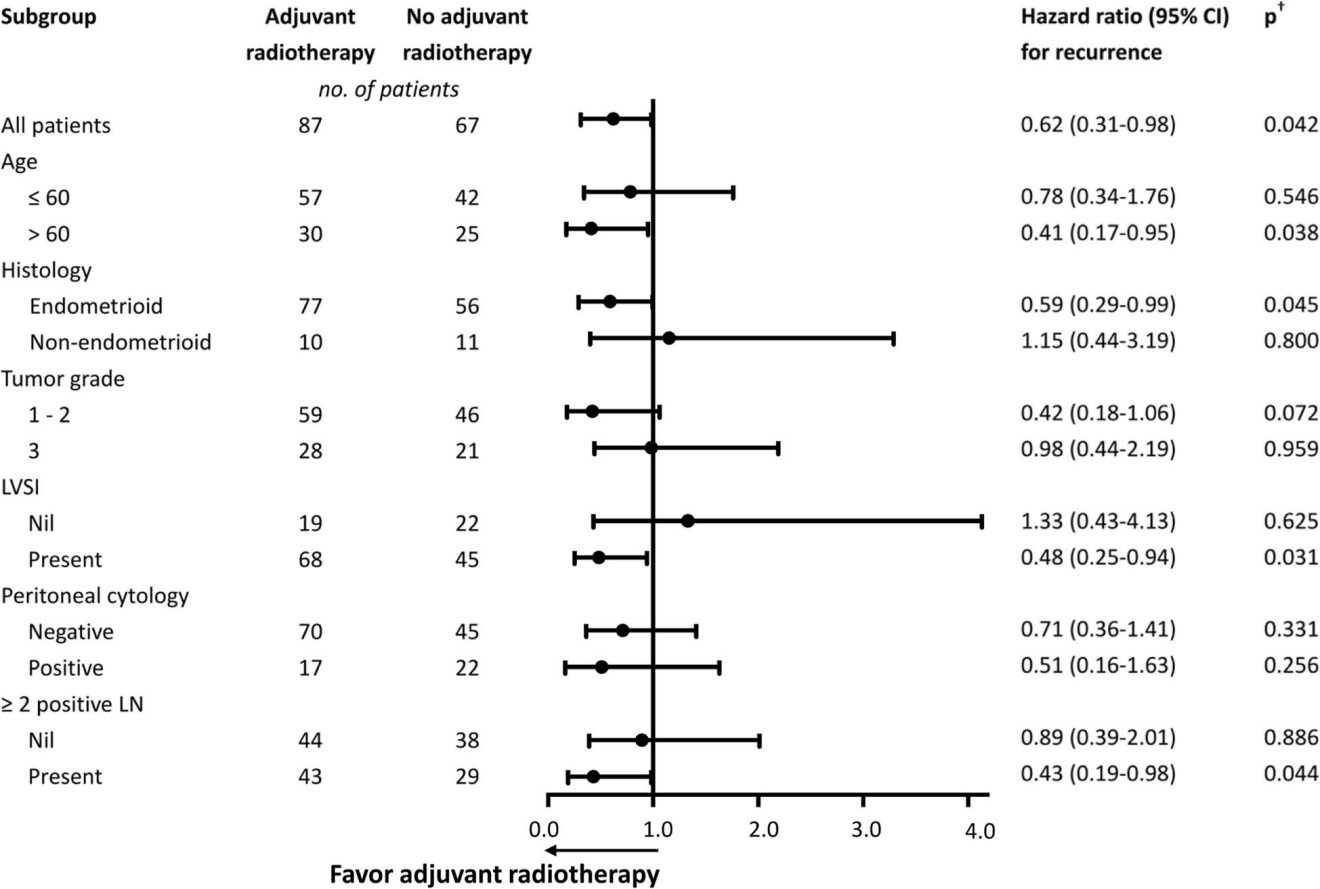
Subgroup analysis of prognostic factors for recurrence-free survival in patients with stage III endometrial cancer (*n* = 154). The hazard ratios and 95% confidence intervals were calculated using the Cox proportional hazards regression model. LN, lymph node; LVSI, lymphovascular space invasion

A subgroup analysis was performed to identify patients who might most benefit from adjuvant radiotherapy (Fig. 3). Adjuvant radiotherapy alone or combined with chemotherapy decreased the risk of tumor recurrence among patients aged > 60 years (*p* = 0.038) as well as those with endometrioid histology (*p* = 0.045), lymphovascular space invasion (*p* = 0.031), and ≥ 2 positive lymph nodes (*p* = 0.044). However, adjuvant radiotherapy did not significantly reduce the risk of recurrence among patients with grade 3 tumors or those with positive peritoneal washing cytology.

## Discussion

To our knowledge, our study is the first to emphasize the value of modern adjuvant radiotherapy, including IMRT and VMAT, on clinical survival specifically in women with optimally resected stage III endometrial cancer. We found that modern adjuvant radiotherapy alone or combined with chemotherapy may be beneficial for patients aged > 60 years as well as those with endometrioid histology, lymphovascular space invasion, and ≥ 2 positive lymph nodes.

In our present study, adjuvant radiotherapy by modern radiotherapy techniques (IMRT and VMAT) led to low incidence of acute (25%) and chronic (7%) grade ≥ 2 gastrointestinal toxicity; by contrast, the aforementioned studies on conventional radiotherapy reported high acute (45%) and late (23%) grade ≥ 2 gastrointestinal toxicity [11]. These data demonstrated that the use of modern radiotherapy techniques (IMRT and VMAT) effectively reduced the dose of radiation in the bowel with a clinical benefit of decreasing acute and late gastrointestinal toxicity [17, 20].

The use of adjuvant radiotherapy for patients with lymph node-positive endometrial cancer is under investigation. Increasing evidence shows that adjuvant radiotherapy improves locoregional control and (potentially) survival not only in patients with early-stage endometrial cancer who have high-risk features, but also in those with advanced-stage endometrial cancer [6, 24–26]. Our data are consistent with previously published studies that showed adjuvant radiotherapy to be effective for patients with node-positive endometrial cancer. Schmid et al. [26], who analyzed 943 patients with (surgically staged) stage III node-positive uterine cancer from the United States’ Surveillance Epidemiology and End Results database, concluded that adjuvant radiotherapy was associated with a significant survival benefit in women with node-positive endometrioid uterine cancers. Brown et al. [24] investigated 116 patients with (surgically staged) stage IIIC endometrial cancer and demonstrated the benefits of adjuvant radiotherapy for those with positive para-aortic lymph nodes and those with ≥2 positive nodes. Secord et al. [25] analyzed 265 patients with stage IIIC disease and concluded that adjuvant radiotherapy alone or combined with chemotherapy improved survival. IMRT and VMAT allow for simultaneous integral boosts to the extranodal extension areas or enlarged unresected lymphadenopathies, and provide accurate positioning and robust radiation doses that produce improved locoregional control and longer disease-free survival.

Several published data suggest that patients with stage III endometrial cancer might benefit from a combination radiotherapy-chemotherapy approach. According to Wang et al. [3], who analyzed 8738 patients from the United States’ National Cancer Database with (surgically staged) stage III uterine cancer who had received adjuvant treatments, concluded that chemoradiotherapy was associated with superior survival outcomes compared to monotherapy. Studies by Kuku et al. [4], Marchetti et al. [5], and Lee et al. [27] of 90, 82, and 66 patients with stage IIIC endometrioid adenocarcinoma, respectively, all concluded that sequential chemoradiotherapy improved patient survival. Most of these data were retrospective and the modalities used were conventional two- or three-dimensional conformal radiotherapy that was associated with insufficient target coverage, inadequate sparing of the organs at risk, and inability to provide a simultaneous integrated boost to the extranodal extension areas or to enlarged unresected lymphadenopathies; all these limitations would compromise the efficacy of radiotherapy.

The prospective GOG 122 randomized controlled trial investigated surgically resected patients with FIGO stage III or IV who received adjuvant whole abdomen irradiation or adjuvant chemotherapy [28] and concluded that chemotherapy was superior to whole abdomen irradiation given that the 5-year OS was 50% in the former group and 38% in the latter group. The GOG 258 trial further investigated patients with surgically resected FIGO stage III or IV who received adjuvant chemotherapy either with or without radiotherapy [9], and concluded that chemotherapy plus radiotherapy (5-year OS, 59%) did not produce superior outcomes than did chemotherapy alone (5-year OS, 58%). However, the radiotherapy administered in the aforementioned trials was conventional and had suboptimal sparing of the organs at risk; this may have underestimated the role of adjuvant radiotherapy. The PORTEC-3 trial investigated radiotherapy alone or with chemotherapy in patients with surgically resected high-risk endometrial cancer [8] and concluded that stage III patients might benefit from the combined approach, as the 5-year OS was 78% in the chemoradiotherapy group and was 68% in the radiotherapy-only group. Our data were in agreement with those of the PORTEC-3 trial, as we showed that modern adjuvant radiotherapy alone or combined with chemotherapy may benefit patients with stage III endometrial cancer.

A therapeutic alternative is brachytherapy, however, although it is considered an integral part of treatment for gynecologic cancers, modern radiotherapy methods (IMRT and VMAT) have shown advantages as substitutes, where brachytherapy is neither possible nor available. In patients who underwent hysterectomy but are contraindicated for, or decline brachytherapy on the vaginal cuff, a simultaneous integrated boost with IMRT or VMAT can be proposed as a therapeutic alternative [29, 30]. Furthermore, given the advantage of the noninvasiveness of radiotherapy generally, modern radiotherapy is considered a winning treatment option for elderly patients or patients unfit for surgery or chemotherapy in order to improve their survival and quality of life [16, 31]. In addition, the advantage of precise high dose targeting with minimum harm to the neighboring organs by IMRT and VMAT allows for a risk-adapted dose prescription, where a boost dose to documented extranodal extension or enlarged unresected lymphadenopathy is technically feasible and clinically effective for higher locoregional control [17]. This information echoes with our results that older patients as well as those with endometrioid histology, lymphovascular space invasion, and ≥ 2 positive lymph nodes benefited the most from adjuvant radiotherapy.

Although accumulating studies of modern radiotherapy methods (IMRT and VMAT) show their superiority to conventional radiotherapy techniques, their limitations include high cost, increased staff burden, longer time for treatment planning, and risks of marginal misses [12]. Our study had several limitations, including its retrospective design. The patient population was heterogeneous in terms of stage subgrouping, tumor histology, and characteristics. Patients did not receive uniform chemotherapy nor multimodality treatments. The median follow-up duration was 44 months; as such, longer follow-up times may be required to further investigate patient outcomes.

## Conclusion

Our data support the indication of adjuvant treatment for women with optimally resected stage III endometrial cancer despite the abovementioned limitations. In the era of modern radiotherapy, adjuvant radiotherapy alone or in combination with chemotherapy was found to be associated with improved survival and may be particularly beneficial for patients > 60 years of age as well as those with endometrioid histology, lymphovascular space invasion, and ≥ 2 positive lymph nodes. These findings may be further investigated in prospective clinical trials.

## Supplementary information

**Additional file 1: Supplementary Figure 1**. Survival in patients with optimally resected stage III endometrial cancer based on whether or not adjuvant treatment was performed. Recurrence-free survival (RFS) (a) and overall survival (OS) (b) of patients based on whether or not adjuvant treatment was performed. *p*-values were determined using Kaplan-Meier log-rank tests.

## Data Availability

The data that support the findings of this study were used under license from the National Taiwan University Hospital Healthcare System database, and so are not publicly available. Limited data are available from the corresponding author on reasonable request.

## Abbreviations

EBRT: External beam radiotherapy
IMRT: Intensity-modulated radiation therapy
VMAT: Volumetric modulated arc therapy
FIGO: International Federation of Gynecology and Obstetrics
RFS: Recurrence-free survival
OS: Overall survival
