# Supplementary figures and images for: Impact of adjuvant radiotherapy on the survival of women with optimally resected stage III endometrial cancer in the era of modern radiotherapy: a retrospective study
Source: Radiat Oncol. 2020 Apr 6;15:72. doi: 10.1186/s13014-020-01523-5 (PMC7137232; doi:10.1186/s13014-020-01523-5)

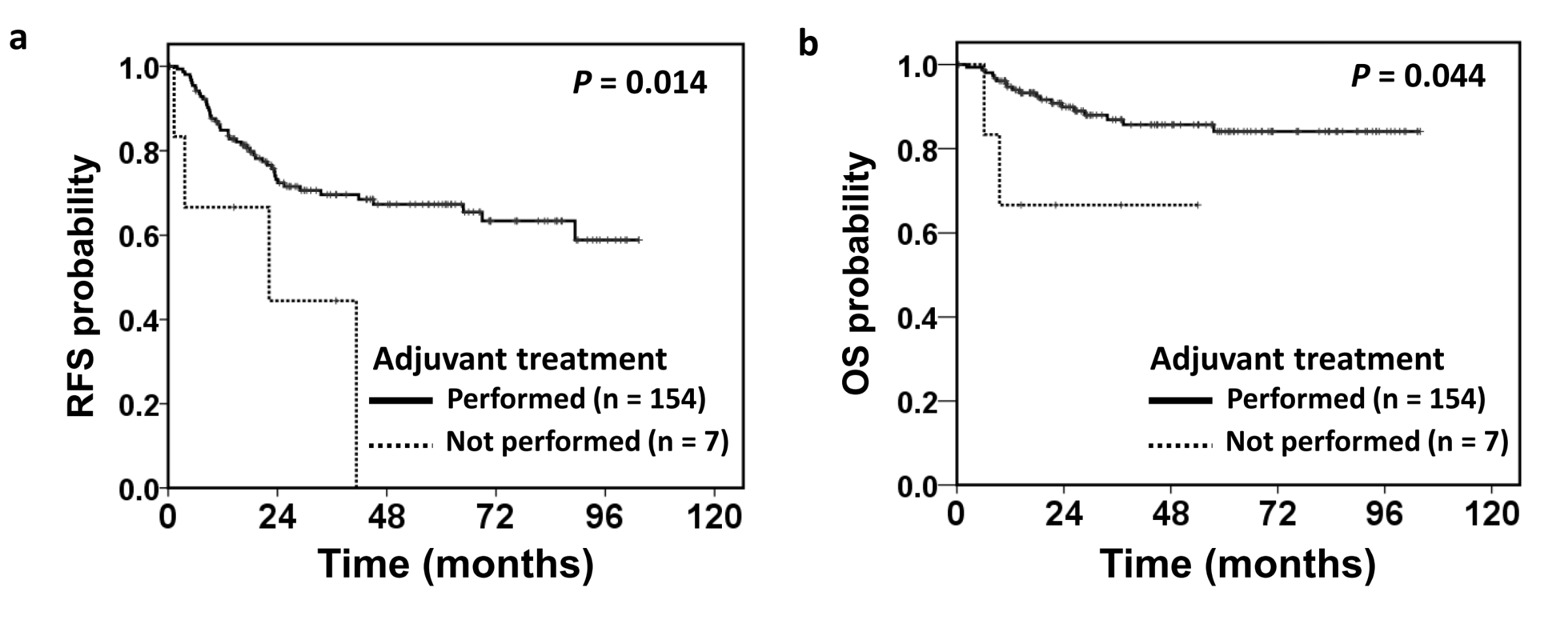

Supplement: Supplementary file 1 — Additional file 1: Supplementary Figure 1. Survival in patients with optimally resected stage III endometrial cancer based on whether or not adjuvant treatment was performed. Recurrence-free survival (RFS) (a) and overall survival (OS) (b) of patients based on whether or not adjuvant treatment was performed. p-values were determined using Kaplan-Meier log-rank tests. [file 13014_2020_1523_MOESM1_ESM.tiff]
